# Fusing Bluetooth Beacon Data with Wi-Fi Radiomaps for Improved Indoor Localization

**DOI:** 10.3390/s17040812

**Published:** 2017-04-10

**Authors:** Loizos Kanaris, Akis Kokkinis, Antonio Liotta, Stavros Stavrou

**Affiliations:** 1 Department of Electrical Engineering, Eindhoven University of Technology, Eindhoven 5600 MB, The Netherlands; a.kokkinis@tue.nl (A.K.); a.liotta@tue.nl (A.L.); 2 Faculty of Pure and Applied Sciences, Open University of Cyprus, Nicosia 2252, Cyprus; stavros.stavrou@ouc.ac.cy

**Keywords:** indoor positioning, indoor localization, fingerprint, bluetooth low energy (BLE), Internet of Things (IoT), Body Sensor Networks (BSN), positioning algorithms

## Abstract

Indoor user localization and tracking are instrumental to a broad range of services and applications in the Internet of Things (IoT) and particularly in Body Sensor Networks (BSN) and Ambient Assisted Living (AAL) scenarios. Due to the widespread availability of IEEE 802.11, many localization platforms have been proposed, based on the Wi-Fi Received Signal Strength (RSS) indicator, using algorithms such as *K*-Nearest Neighbour (KNN), Maximum A Posteriori (MAP) and Minimum Mean Square Error (MMSE). In this paper, we introduce a hybrid method that combines the simplicity (and low cost) of Bluetooth Low Energy (BLE) and the popular 802.11 infrastructure, to improve the accuracy of indoor localization platforms. Building on KNN, we propose a new positioning algorithm (dubbed i-KNN) which is able to filter the initial fingerprint dataset (i.e., the radiomap), after considering the proximity of RSS fingerprints with respect to the BLE devices. In this way, i-KNN provides an optimised small subset of possible user locations, based on which it finally estimates the user position. The proposed methodology achieves fast positioning estimation due to the utilization of a fragment of the initial fingerprint dataset, while at the same time improves positioning accuracy by minimizing any calculation errors.

## 1. Introduction

In the Internet of Things (IoT) and Body Sensor Networks (BSN), several scenarios envision the integration of various wireless technologies that will provide services based on the user behaviour [1,2]. Typical scenarios enabled by BSN technologies, include m-Health, e-Sport, e-Fitness, and e-Wellness. In all these applications, numerous programmable wireless sensors, with enhanced capabilities, are combined and configured in order to directly monitor several parameters in a non-invasive way [3]. A comprehensive and systematic review of the state-of-the-art techniques on multi-sensor fusion in the BSN research area is provided in [4]. Nowadays, where user profiling is very important, user localization and tracking are instrumental to a broad range of such services and applications [5,6,7,8]. User localization, in areas where the Global Positioning System (GPS) is not available, is typically achieved by utilizing several wireless communication technologies (Wi-Fi, Bluetooth, Long Term Evolution (LTE), Zigbee, Visual Light Communication (VLC), etc.).

To improve localization accuracy, the research community investigated more sophisticated solutions that are often sought through hybrid approaches. Such methods involve the combination of different parameters such as Time of Flight (TOF), Received Signal Strength (RSS), RSS Difference (RSSD), Angle of Arrival (AOA), Time Of Arrival (TOA), Time Difference Of Arrival (TDOA), etc. Wireless Indoor Positioning Techniques have been surveyed in [9,10,11,12], while key approaches on Visual Light Positioning Systems (VLP) were discussed in [13]. Among all of these, fingerprint-based positioning is one of the most popular indoor localization techniques implemented by Real-Time Localization Systems (RTLS).

Localization applications implementing fingerprint methods can find use in malls, for navigating people and providing marketing alerts; in hospitals, for monitoring patients, doctors and critical equipment; in logistics, for tracking assets and optimizing empty spaces in ports or inland storages; and in homes, for Ambient Assisted Living (AAL) services [14]. The main advantage of fingerprinting techniques is that they can utilize the existing wireless communication infrastructures, without the need to deploy any additional, specialized equipment. The dataset of fingerprints, called a “radiomap”, is the basis behind the positioning algorithms. Radiomaps can be generated rapidly and at a relatively low cost, particularly when a deterministic radio propagation simulator is used, instead of performing costly and lengthy measurement campaigns [15]. Fingerprinting requires both an offline and an online phase. During the offline phase, the radiomap is generated by recording the Access Points (AP) RSS values (that can be either measured or obtained through a simulator) for each location in the area of interest. Calibration and training techniques are usually used during this phase, in order to improve the quality of the radiomap [16]. During the online phase, the Mobile Station (MS) performs network discovery as well as real-time RSS measurements. Different positioning algorithms are then applied in order to identify the best match between the observed RSS fingerprint and the respective mean value of the fingerprints recorded during the offline phase. An overview of the most significant fingerprint-based methods is provided in [17].

New opportunities in user’s localization and indoor positioning have emerged with the introduction of Body Sensor Networks and smart devices that are able to support several IEEE technologies like 802.15.4 and IEEE 802.11 technologies. A promising research direction is based on Visual Light Positioning (VLP). In this case, positioning accuracy can be achieved only through dense smart light grids, thus incurring excessive infrastructure costs [12]. In addition, mobile users need to have a line-of-sight with smart lights while their smart devices have to support the required technology and provide the necessary processing power [18]. On the other hand, by combining existing well-established Wi-Fi positioning systems with Bluetooth Low Energy (BLE) i-beacons, an excellent opportunity is created to enhance the user’s localization accuracy. This approach can make fingerprinting even more favourable, particularly in smart homes, since localization accuracy can be pursued by deploying only a small number of low-cost BLEs on top of the existing Wi-Fi infrastructure. This is, in fact, the methodology pursued in this paper, whereby we introduce a new method to combine BLE with Wi-Fi fingerprint positioning, in order to significantly improve the achieved localization accuracy.

The rest of the paper is organised as follows: Section 2 presents related work on radio RSS fingerprint-based methods and summarizes the BLE technology. We introduce our method in Section 3, explaining the rationale for the formulation of the i-KNN algorithm. Section 4 and Section 5 describe the testing methodology and performance evaluation that is based on both simulations and actual measurements. Finally, Section 6 draws the conclusions and makes suggestions for future work.

## 2. Related Work

### 2.1. Radio RSS Fingerprint-Based Indoor Positioning Methods

RSS fingerprint–based positioning methods are utilized in various wireless technologies (IEEE 802.11, IEEE 802.15.4, etc.). The main advantage of these methods is the relatively low complexity of the positioning system, which utilizes existing infrastructure, rather than requiring the deployment of specialized equipment. The positioning algorithms retrieve the RSS at the user location and implement either deterministic or probabilistic methodologies to estimate the actual user location. The concept that lies behind both methodologies is common; searching a database of fingerprints and identifying one or more positions whose RSS signature has the *highest similarity* with the observed one. More specifically, *deterministic* positioning methods estimate location $\hat{\ell }$ as a convex combination of the *K* reference locations with the shortest distance between ${\bar{r}}_{i}$ and *s* in the *n*-dimensional space [19,20,21]. The aforementioned statement is mathematically expressed with the following equation:

(1) $$\hat{\ell }=\sum_{i=1}^{K}\left(\frac{w_{i}}{\sum_{j=1}^{K}w_{j}}\ell_{i}^{\prime }\right).$$

The set $\left\{\ell_{1}^{\prime },\ldots ,\ell_{l}^{\prime }\right\}$ denotes the ordering of reference locations with respect to increasing distance $D_{i}$, which is measured between the respective database fingerprint ${\bar{r}}_{i}$ and the observed measurement during positioning *s*, i.e., $D_{i}=∥{\bar{r}}_{i}-s∥$. The distance can be calculated using standard norms, such as the Manhattan (1-norm) [22], the Euclidean (2-norm) [23] or the Mahalanobis norm [24]. Focusing on the Euclidean distance, $D_{i}$ can be expressed by the following equation:

(2) $$D_{i}=\sqrt{\sum_{j=1}^{N}{\left({\bar{r}}_{ij}-s_{j}\right)}^{2}}.$$

The non-negative weight coefficient $w_{i}$ in Equation (1) represents a value that can be allocated to each reference location in the radiomap and differentiates its weight, hence its importance from other fingerprints. In other words, the value of this $w_{i}$ coefficient may vary in a way that each fingerprint influences differently the positioning estimation. In such a case of weight allocation, Equation (1) expresses the Weighted *K*-Nearest Neighbour (WKNN) algorithm [22]. A typical value for $w_{i}$ can be the inverse of $∥{\bar{r}}_{i}-s∥$. Simplifying the aforementioned algorithm, it can be assumed that equal weights are allocated to all utilized fingerprints. Such an assumption results in the elimination of $w_{i}$ and the equation is converted to the *K*-Nearest Neighbour (KNN) method. Finally, setting K=1, the formula leads to the simple Nearest Neighbour (NN) method [23,25]. According to [22,23], KNN and WKNN methods provide higher positioning accuracy compared to the NN, for K=3 and K=4. On the other hand, NN seems to perform satisfactorily and provides equally good results in scenarios with high density RSS radiomaps [19]. More complex deterministic algorithms are discussed in literature, such as the linear discriminant analysis [26] and the Database Correlation Method (DCM) [27]. They generally claim better localization accuracy but at a higher computational cost.

In *probabilistic* methods, location *ℓ* can be estimated by calculating and maximising the conditional posterior probabilities $p(\ell_{i}|s)$, where i=1,…,l, given an observed point *s* and a reference radiomap of *l* fingerprints.

The posterior probability $p(\ell_{i}|s)$ is obtained by applying Bayes’ rule:

(3) $$p\left(\ell_{i}|s\right)=\frac{p\left(s|\ell_{i}\right)p\left(\ell_{i}\right)}{\sum_{i=1}^{l}p\left(s|\ell_{i}\right)p\left(\ell_{i}\right)},$$

where $p(s|\ell_{i})$ is a conditional probability calculated through statistics at the survey stage and $p(\ell_{i})$ is the a priori probability, a weighting factor based on the probability distribution of the observation over the reference position candidates included in the fingerprint database. In case of no prior knowledge, this *prior* is assumed to be unity, meaning that all fingerprint candidates have equal a priori probability. Probabilistic methods have been used in the Maximum A Posteriori (MAP) approach [28] and the Minimum Mean Square Error (MMSE) approach [29] to estimate the expected value of *ℓ*.

In a continuous effort for improving localization accuracy in fingerprint-based systems, the research community investigated the fusion of data retrieved by the indoor mapping of buildings. Towards this direction, Evennou et al. in [30] proposed the use of particle filters to make use of the inherent structure of indoor environments, while, more recently, Kokkinis et al. in [21], proposed a method of imposing map-constraints into the positioning algorithms in the form of a-priori knowledge. Finally, the fusion of information received by several sources was examined in [31], while the incorporation of wearable devices for indoor localization was also investigated in [14].

### 2.2. BLE and the iBeacon Technology

BLE was introduced as part of Bluetooth 4.0 specifications, allowing the devices to support both BLE and classic Bluetooth protocols simultaneously [32]. The power efficiency of Bluetooth with low energy functionality was especially created for IoT applications. It allows devices to run for long periods on extremely low power sources, such as coin-cell batteries or energy-harvesting devices.

BLE operates at 2.4 GHz and uses Gaussian Frequency Shift Key (GFSK) modulation in 40 channels of 2 MHz. Three of the channels, called “advertising channels”, are used to ensure connectivity with other nodes, while the remaining 37 are the “data channels”. BLE has a range of around 100 m in an outdoor environment, a maximum data rate of 1 Mbit/s and an application throughput up to 305 kbit/s [32]. Finally, it supports point-to-point and mesh networks.

iBeacon was developed by Apple (Cupertino, CA, USA) in order to provide a higher level of location awareness, by utilizing the BLE technology. iBeacon is a cross platform technology for both Android and iOS devices that are able to support the BLE standard [33]. Devices, acting as beacons, generate iBeacon advertisements through which they establish a region around them. Android and iOS mobile devices receiving the advertisements can determine the entrance and exiting borders from each Beacon’s region, can estimate the nearest beacon and can approximate the distance between the two devices. The aforementioned advertisements contain three identifying fields, as described in [34]:

- **UUID:** Universally Unique Identifier is a 128-bit integer used as an ID for all beacons in an application;
- **Major:** is a 16-bit integer, used to differentiate Beacons with the same UUID;
- **Minor:** is a 16-bit integer used to further differentiate Beacons that have the same UUIDs and Major values.

Due to their design philosophy, iBeacons are flexible in deployment and can be used in mobile objects or to temporarily define a region and subregions.

The possibilities introduced by BLE and iBeacon technologies in indoor positioning are currently a topic of investigation from the research community. The authors of [35] proposed a software framework that can be used to automate IoT applications based on the proximity triggered by AltBeacon devices. AltBeacon is an open and interoperable specification that defines the format of the advertisement message that BLE proximity beacons broadcast. In [36], InLoc is introduced, which is a positioning and tracking system using commercial mobile devices, with a navigation (routing) capability. With InLoc, the authors propose, among others, a method for independent fusion of location information from phone Inertial Measurement Unit (IMU) sensors and BLE beacons. In [37], the authors propose a solution for the creation of study groups in future smart libraries, featuring a smart-phone application to create study groups and a hybrid BLE and Wi-Fi indoor positioning system. Their hybrid indoor positioning system calculates two probable user locations every time, utilizing each technology separately, compares the estimated conditional probabilities and selects the most reliable. Following a different approach, researchers in [38] assume a very dense IoT environment with BLE compatible devices and propose an Iterative Weighted KNN (IW-KNN) indoor localization method based on RSS of the BLE, which has a low power consumption and hence a long life expectancy. Finally, a combination of Wi-Fi and BLE fingerprints was implemented in [39], utilizing the conventional WKNN algorithm. The test case included the deployment of 17 Estimote BLEs (Estimote Inc., New York, NY, USA) on top of the existing Wi-Fi network, in a study area of 52 m × 43 m. Positioning was performed by utilizing both BLE and Wi-FI RSS fingerprints, resulting in a 23% accuracy improvement of the RTLS system.

## 3. Proposed Approach

Our approach is to gather general localization data from BLE devices deployed in an IoT environment and use them to optimize the data retrieved from existing popular and low cost IEEE 802.11 RSS fingerprint-based indoor positioning systems, in order to improve the provided positioning accuracy. Implementing this concept, a new enhanced KNN positioning algorithm was developed (i-KNN), which is able to filter the initial Wi-Fi fingerprint dataset (radiomap), taking into consideration the proximity of the RSS fingerprints to the BLE devices. By choosing to filter the initial dataset instead of simply combining BLE and Wi-Fi fingerprints, i-KNN utilizes an optimised small size subset of possible user locations for the final position estimation.

**Figure 1:**
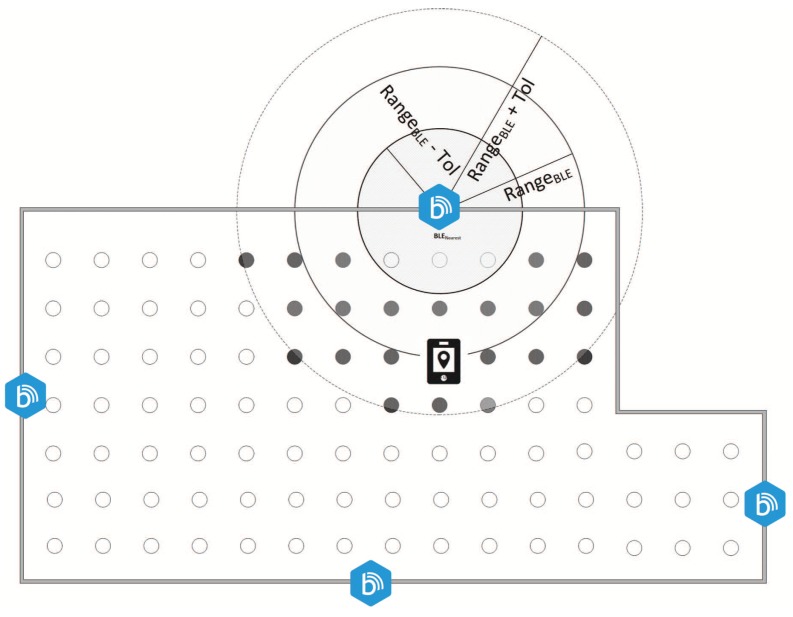
Concept of the proposed i-KNN algorithm: Bluetooth Low Energy (BLE) utilization for Wi-Fi radiomap subset generation.

The i-KNN algorithm uses the data transmitted from the i-Beacons concerning the estimated distance between a BLE device and the mobile user (mobile device or body sensor), as well as the information referring to the nearest i-Beacon of the whole BLE network. These data are used as an input to the filtering processes of the i-KNN in order to roughly estimate a probable area *A* which encloses the user’s position. The aforementioned donut-shape area, *A*, is formulated between a minimum and a maximum radius ($R_{BLE_{min}}$ and $R_{BLE_{max}}$) measured from a center point, where the BLE device is located. The $R_{BLE_{min}}$ and $R_{BLE_{max}}$ values are calculated, taking into consideration a predefined tolerance (Tol) parameter, which accommodates any positioning error factors. As illustrated in Figure 1, area *A* is then used to screen the number of candidate fingerprints, down to a subset ($S:\left\{\ell_{1},\ldots ,\ell_{k}\right\}$) extracted from the initial IEEE 802.11 fingerprint dataset ($D:{\left\{\ell_{1},\ldots ,\ell_{j}\right\}}_{Wi-Fi}$). The filtered fingerprint data subset *S* is finally used as the optimized input, to typical indoor positioning algorithms (in our case the KNN). The proposed methodology serves two purposes: firstly, achieving fast positioning estimation due to the utilization of a fragment of the initial fingerprint dataset and secondly achieving improved positioning accuracy by constraining any possible calculation errors within a very specific area *A*, where the user is actually located. The latter is achieved due to the inherited short range of IEEE 802.15 and its capability to identify the nearest i-beacon device to each mobile user. For clarity, a self-explanatory pseudocode of the i-KNN filtering algorithm used to calculate the subset $S:\left\{\ell_{1},\ldots ,\ell_{k}\right\}$ is presented in Algorithm 1 and explained in Subsection 3.1.

### 3.1. i-KNN Algorithm Explanation

The filtering algorithm receives as an input the Wi-Fi radiomap $D:{\left\{\ell_{1},\ldots ,\ell_{j}\right\}}_{Wi-Fi}$ and BLE locations $B:\{BLE_{1}\ldots ,BLE_{i}\}$. A procedure named Fingerprints to BLE distance pre-calculates in advance distances between BLE devices and all fingerprint locations for easier estimation of the final output, which is subset *S*. Calculated distances are given at the form of a matrix $L:\{r_{\ell_{1},BLE_{1}}\ldots ,r_{\ell_{j},BLE_{i}}\}$. During the real-time positioning estimation, the algorithm initially scans for traceable BLEs by running BLE Discovery (B) procedure, retrieves their parameters and sorts them accordingly based on their Received Signal Strength. Upon retrieval of BLE information, i-KNN selects the nearest BLE device and utilizes the distance information broadcast by the beacon ($Range_{BLE_{i}}$) to additionally calculate the Tolerance Tol level and designate the donut-shape area, *A*, shown in Figure 1. Tol can be either a constant number as in this research work, or can be dynamically calculated as a percentage b% of the distance information: $\pm Range_{BLE_{i}}b\%$. In the latter case, the closer the MS user is to the BLE, the smaller the Tol will be, and hence a smaller optimized dataset will be selected. This methodology is a more optimized option, since the BLE-MS user distance calculation is more reliable at smaller distances. In either case, upon calculation of area *A*, the optimized dataset, $S:\left\{\ell_{1},\ldots ,\ell_{k}\right\}$, is extracted by utilizing the pre-calculated distances retrieved from $L:\{r_{\ell_{1},BLE_{1}}\ldots ,r_{\ell_{j},BLE_{i}}\}$ matrix. Finally, instead of utilizing the heavy initial radiomap *D*, the much smaller dataset *S* feeds the typical KNN methods in order to estimate the location of the MS user.

### 3.2. i-KNN in Pseudocode Form

For clarity reasons, the i-KNN is presented below in the form of a pseudo-code:

**Algorithm 1:**
:i−KNN
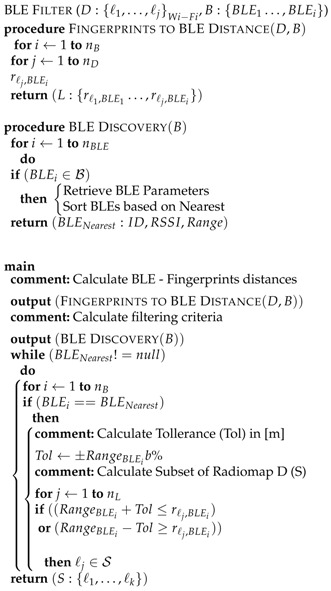


## 4. Test Environment

**Figure 2:**
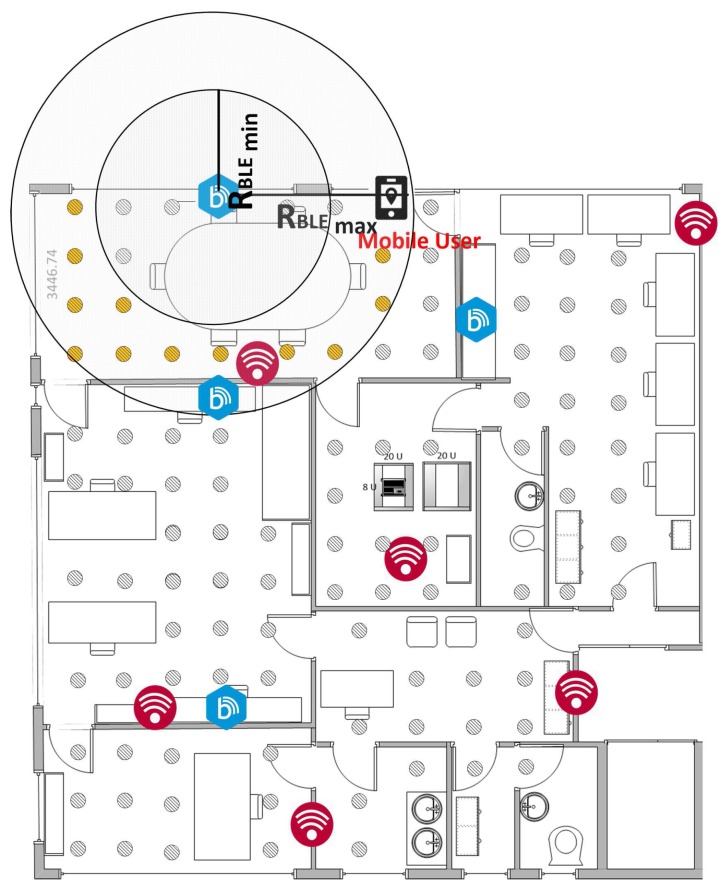
Combined BLE and Wi-Fi fingerprint based indoor positioning.

The i-KNN algorithm was tested by combining actual measurements and simulations. In order to accomplish this task, two independent networks were deployed in an indoor environment of approximately 160 m${}^{2}$. The IEEE 802.11 wireless network is composed of six D-Link 802.11 APs, the allocation of which is shown in Figure 2. The BLE network consisted of four IEEE 802.15 BLE *Estimote* devices. Each device was located in a different room of the test environment as illustrated in Figure 2. Two radiomaps were then generated: the first one through a measurement campaign; and the second one through *TruNET wireless*, a full 3D Ray tracing simulator [40].

### 4.1. Radiomap from Actual Measurements

During the measurement campaign, fingerprints collected at 110 equally-spaced (1 m spacing) locations, at a constant height of 90 cm. At every measurement point, 30 district RSS samples (1 sample/sec) were recorded using an application developed for Android-based MS devices. The RSS values recorded in the radiomap ranged from –99 dBm to –34 dBm. During the network discovery procedure, the system recorded data from 24 APs from other neighbouring networks, hence a filtering procedure was implemented prior to the finalization of the dataset.

### 4.2. Simulated Radiomap

The second radiomap was generated by using a deterministic 3D Ray Tracing propagation model, employed by *TruNET wireless* [40]. The building structure and the different furniture were configured using material constitutive parameters as obtained from literature [41]. They were finally calibrated as presented in Table 1 in order to better match the MS device characteristics. A detailed analysis of the Ray tracing calibration procedure can be found in [42]. The same 110 measurement points, were defined as receiver cells. Finally, the six APs were configured as per the characteristics provided by the manufacturers.

### 4.3. BLE Filtering

Initially, testing measurements were performed by implementing the simple KNN algorithm (K=4) on an IEEE 802.11 typical radiomap, in order to retrieve reference benchmark values, at 12 randomly selected locations. This strategy allowed the authors to perform result analysis that could objectively depict the improvement on the localization accuracy of the positioning algorithms under study. Objective evaluation is achieved through the use of benchmark values by maintaining the external environment unmodified during the experiment execution, keeping the testing locations constant and performing the measurements under static conditions (no mobility allowed). After the retrieval of the benchmark values, the proposed i-KNN filtering algorithm was implemented for two different scenarios and test measurements were once again performed at the same 12 locations. The first scenario assumed that only one i-Beacon device existed in the study area, while the second scenario took into consideration that all four i-Beacons were deployed as per Figure 2. In all test cases, an average number of 28 samples was collected, in order to ensure that the sample size was large enough for the normal distribution statistical parameters to apply. In other words, the calculated standard deviation values, and consequently any extracted confidence levels, were statistically acceptable and could provide reliable result analysis [16]. The user orientation was also examined for investigating the body presence effect. Finally, the influence of i-Beacon number and location was also examined by varying the number of active BLEs. The variation of the localization error in the above cases defined the maximum tolerance Tol parameter value at Tol = 2 m. In this way, it was ensured that the user’s actual location was falling within the candidate fingerprints chosen in the optimized dataset. Our findings were consistent with [38], where BLE RSS indication fluctuated up to 10 dB. Error factors covered by the introduction of the Tol parameter included the actual number of active BLEs, user/device orientation, body and multipath effects.

## 5. Performance Evaluation

**Figure 3:**
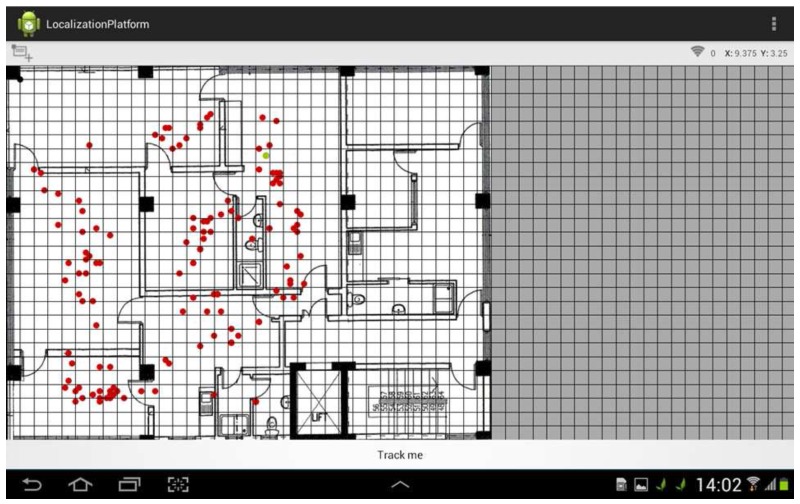
ϕmap and localization platform.

For the practical implementation and testing of the positioning algorithms, an Android fingerprint-based localization platform (ϕmap) was developed, providing configuration capabilities for several parameters related with KNN and i-KNN algorithms. The most important parameters include K value, number of samples recorded per point, time interval between each sample and Tolerance (Tol) value. ϕmap also provides the possibility to select between different radiomaps (generated by both actual measurements or simulations) and to upload a 2D blueprint of the study area for user friendly visualization of the user position. A snapshot of the application is illustrated in Figure 3.

The Wi-Fi Indoor Positioning system was fully deployed, and tests were performed at 12 randomly selected locations as described previously for the benchmark (Wi-Fi only) case and the two hybrid (Wi-Fi and BLE) scenarios. Data retrieval was performed with the MS user to be static, in order to retrieve a statistically adequate number of samples allowing a valid statistical analysis. However, both ϕmap platform and i-KNN algorithm can support real-time moving MS users, within the typical constraints of the KNN algorithm. In other words, a time delay may occur on tracking a fast moving user, depending on the K value and the number of samples retrieved per point. The experimental results concerning the positioning error of the platform utilizing only the IEEE 802.11 radiomap and implementing the typical KNN algorithm, is presented in Table 2. An average positioning error of *e* = 4.05 m with standard deviation σ = 2.13 is achieved for the specific environment under study. The findings of the benchmark case are aligned with the performance of other typical Wi-Fi indoor localization systems, a comparison of which is illustrated in Table 3. A general conclusion extracted from the aforementioned table is that typical RSS fingerprint-based positioning systems can provide an accuracy ranging from 3 m to 7 m, practically meaning a room level designation.

Test results for the first hybrid scenario (existence of a single BLE device) are shown in Table 4. The positioning error *e* is improved to 3.07 m with a smaller standard deviation of σ = 1.60, while the utilized fingerprint dataset size is reduced to an average of 67%. At five out of 12 test points, the i-KNN algorithm utilized the total number of fingerprints as included in the initial radiomap, since no BLE signal was identified in these specific locations, due to wall attenuation effects. Although BLE signals could penetrate single plasterboard walls and double glass windows, they were heavily weakened when transmission occurred through cement and brick walls. At those five test points, the i-KNN algorithm did not have any effect and ϕmap operated as a **Wi-Fi only** platform. During the second hybrid scenario, the full deployment of the BLE devices ensured that location information from at least one i-Beacon was retrieved in all 12 test locations. Test results of this scenario are presented in Table 5. A radical improvement of the positioning accuracy and optimization of the utilized dataset occurred. More specifically, the error was reduced to 2.33 m for the same study area, indicating an improvement of 42%. Standard deviation was also significantly reduced to 1.22, depicting the much higher concentration of results around the mean values of positioning results. Additionally, the utilized dataset size was significantly reduced, ranging between 12% and 37%, depending on the test point. Taking into consideration the comparison Table 3, it is observed that the proposed i-KNN algorithm overperforms the typical Wi-Fi localization platforms. As a price for increased accuracy, the RTLS operators need to deploy a number of BLE systems is such a geometry that can cover the maximum area of interest. Obviously, such a deployment depends on the complexity of the indoor environment; open plan spaces require less BLE devices than wall-separated areas.

**Figure 4:**
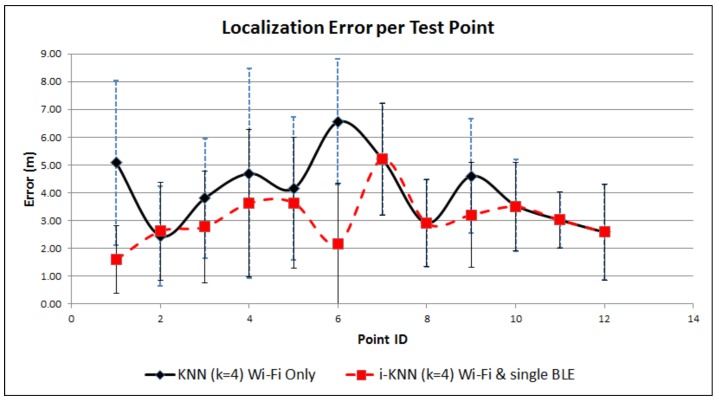
Positioning error comparison: Wi-Fi only vs. single BLE and Wi-Fi.

**Figure 5:**
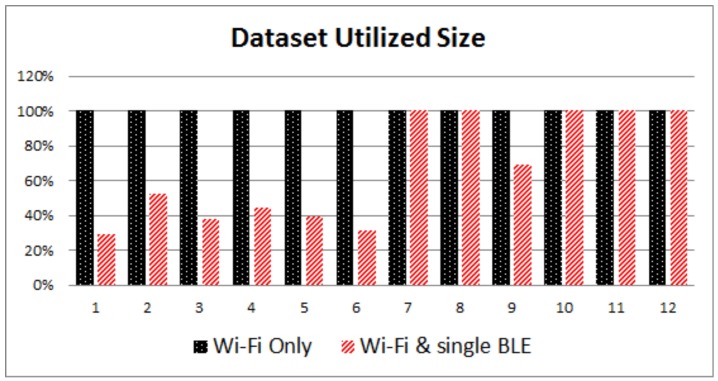
Fingerprint dataset size utilization: Wi-Fi only vs. single BLE and Wi-Fi.

**Figure 6:**
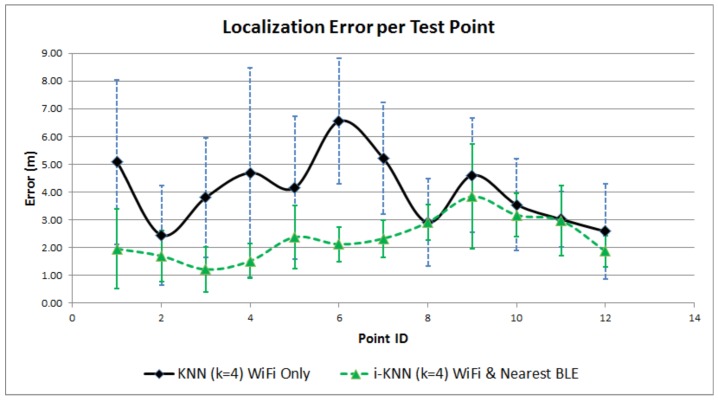
Positioning error comparison: Wi-Fi only vs. nearest BLE and Wi-Fi.

**Figure 7:**
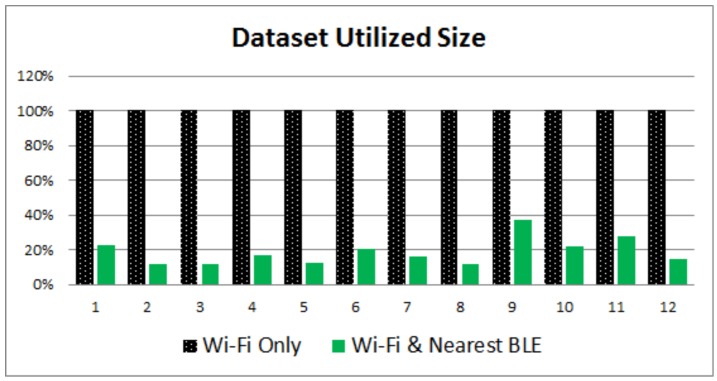
Fingerprint dataset size utilization: Wi-Fi only vs. nearest BLE and Wi-Fi.

Figure 4 and Figure 5 provide a visual performance comparison for the first scenario, while Figure 6 and Figure 7 refer to the outcomes of the second scenario. What is obvious from the graphs is that the combination of Wi-Fi and BLE systems in the proposed i-KNN algorithm constantly outperforms the simple KNN, especially when the BLE deployment is such that it can provide adequate signal coverage in the study area. In such scenarios, accuracy is improved and positioning results fluctuate much less, as indicated by the lower standard deviation. Dataset utilization is optimized to an average of 20% for typical scenarios and can be further improved if the set Tolerance Tol factor is further optimized to a minimum value. Overall, the findings provide hard evidence that the proposed i-KNN algorithm improves the computational and processing requirements, provides faster and more accurate positioning and incurs lower power consumption.

## 6. Conclusions

In this work, a novel positioning algorithm (i-KNN) is proposed, which fuses information available from the emerging low cost BLE technology with popular IEEE 802.11 based fingerprint localization platforms. The new algorithm was tested by combining actual measurements and simulations, and evidence was provided that it significantly improves localization accuracy and computational performance. Localization accuracy for the test case was improved from 4.05 m to 2.33 m. Computational performance was indicated by the reduction of the utilized dataset size, to a range between 12% and 37% of the initial radiomap size. The scalability of the i-KNN algorithm makes it ideal for advancing the performance of existing fingerprint based RTLS systems. BLE devices can be either deployed solely for positioning purposes or can be combined with other uses. Future work includes investigating an optimal BLE deployment and an in-depth analysis of the body effect and sensor orientation, in order to further optimize the indoor localization performance. Computational performance improvements as well as battery saving may also be investigated, utilizing different models of smart devices under intensive use of the i-KNN algorithm versus other hybrid solutions.

## Figures and Tables

**Table 1:** Material constitutive parameters of the test environment.

Material	Electrical Permittivity (F/m)	Loss Tangent
**Concrete**	3.9	0.23
**Wood**	2	0.025
**Brick**	5.5	0.03
**Metal**	1	1,000,000
**Plasterboard**	3	0.067
**Glass**	4.5	0.007

**Table 2:** Positioning error of Wi-Fi Received Signal Strength (RSS) fingerprint-based positioning system.

Test Point	$e_{\mathrm{average}}$ (m)	σ	Radiomap Size (%)	Samples No.
**1**	5.09	2.96	100	19
**2**	2.45	1.79	100	21
**3**	3.81	2.15	100	21
**4**	4.70	3.76	100	20
**5**	4.15	2.56	100	25
**6**	6.55	2.26	100	23
**7**	5.21	2.02	100	16
**8**	2.91	1.56	100	29
**9**	5.61	2.02	100	37
**10**	3.50	1.58	100	50
**11**	3.02	1.01	100	30
**12**	2.58	1.72	100	45

**Table 3:** Positioning error of typical indoor Wi-Fi positioning systems.

System	Accuracy/Error	Methodology	Complexity
**TIX, [43]**	5.4 m	linear mapping of RSS	light algorithm with AP modifications
**EZ, [44]**	2.0–7.0 m	model based	complex algorithm
**SDM, [45]**	3 m	linear mapping of RSS	light algorithm with sniffers
**Zee, [46]**	3 m	RSS fingerprints	combined with Horus or EZ
**LiFS, [47]**	89% *room level*	RSS fingerprints	complex training phase
**WILL, [48]**	86% *room level*	RSS fingerprints	complex mapping of virtual floor
ϕmap, *Wi-Fi only*	4.05 m	RSS fingerprints	light algorithm

**Table 4:** Positioning error of combined BLE (single BLE) and Wi-Fi RSS fingerprint-based positioning system.

Test Point	$e_{\mathrm{average}}$ (m)	σ	Radiomap Size (%)	Samples No.
**1**	1.60	1.22	29	19
**2**	2.62	1.75	53	21
**3**	2.77	2.01	38	21
**4**	3.63	2.65	45	20
**5**	3.64	2.35	40	25
**6**	2.17	2.16	39	23
**7**	5.21	2.02	100	16
**8**	2.91	1.56	100	29
**9**	3.20	1.90	69	37
**10**	3.50	1.58	100	50
**11**	3.02	1.01	100	30
**12**	2.58	1.72	100	45

**Table 5:** Positioning error of combined BLE (all deployed BLEs) and Wi-Fi RSS fingerprint-based positioning system.

Test Point	$e_{\mathrm{average}}$ (m)	σ	Radiomap Size (%)	Samples No.
**1**	1.96	1.44	22	19
**2**	1.70	0.93	12	21
**3**	1.22	0.81	12	21
**4**	1.52	0.62	16	20
**5**	2.38	1.14	12	25
**6**	2.12	0.63	16	23
**7**	2.33	0.67	16	16
**8**	2.91	0.65	12	29
**9**	3.84	1.89	37	37
**10**	3.17	0.79	22	50
**11**	2.97	1.26	28	30
**12**	1.87	0.55	19	45

## Abbreviations

The following abbreviations are used in this manuscript:

AAL	Ambient Assisted Living
AOA	Angle of Attack
AP	Access Point
BLE	Bluetooth Low Energy
BSN	Body Sensor Networks
DCM	Database Correlation Method
DOAJ	Directory of Open Access Journals
GFSK	Gaussian Frequency Shift Key
i-KNN	intelligent KNN
IMU	Inertial Measuring Unit
IoT	Internet of Things
IW-KNN	Iterative Weighted KNN
KNN	K-Nearest Neighbour
LTE	Long Term Evolution
MAP	Maximum A Posteriori
MDPI	Multidisciplinary Digital Publishing Institute
MMSE	Minimum Mean Square Error
MS	Mobile Station
NN	Nearest Neighbour
RSS	Received Signal Strength
RSSD	RSS Difference
RTLS	Real-Time Localization System
TDOA	Time Difference of Arrival
TOA	Time of Arrival
TOF	Time of Flight
VLC	Visual Light Communication
VLP	Visual Light Positioning
WKNN	Weighted K-Nearest Neighbour

## References

[1] Macagnano D., Destino G., Abreu G. Localization with heterogeneous information. Proceedings of the IEEE World Forum on Internet of Things.

[2] Rabadan J., Guerra V., Rodríguez R., Rufo J., Luna-Rivera M., Perez-Jimenez R. (2017). Hybrid Visible Light and Ultrasound-Based Sensor for Distance Estimation. Sensors.

[3] Fortino G., Giannantonio R., Gravina R., Kuryloski P., Jafari R. (2013). Enabling Effective Programming and Flexible Management of Efficient Body Sensor Network Applications. IEEE Trans. Hum. Mach. Syst..

[4] Gravina R., Alinia P., Ghasemzadeh H., Fortino G. (2017). Multi-sensor fusion in body sensor networks: State-of-the-art and research challenges. Inf. Fusion.

[5] Chen M. (2013). Towards smart city: M2M communications with software agent intelligence. Multimedia Tools Appl..

[6] Zhang D., Yang L., Chen M., Zhao S., Guo M., Zhang Y. (2016). Real-Time Locating Systems Using Active RFID for Internet of Things. IEEE Syst. J..

[7] Ji H., Xie L., Wang C., Yin Y., Lu S. (2015). CrowdSensing: A crowd-sourcing based indoor navigation using RFID-based delay tolerant network. J. Netw. Comput. Appl..

[8] Macagnano D., Destino G., Abreu G. Indoor positioning: A key enabling technology for IoT applications. Proceedings of the IEEE World Forum on Internet of Things.

[9] Al Nuaimi K., Kamel H. A survey of indoor positioning systems and algorithms. Proceedings of the 2011 International Conference on Innovations in Information Technology.

[10] Deak G., Curran K., Condell J. (2012). A survey of active and passive indoor localisation systems. Comput. Commun..

[11] Hossain A.M., Soh W.S. (2015). A survey of calibration-free indoor positioning systems. Comput. Commun..

[12] He S., Chan S. (2016). Wi-Fi Fingerprint-Based Indoor Positioning: Recent Advances and Comparisons. IEEE Commun. Surv. Tutor..

[13] Lausnay S.D., Strycker L.D., Goemaere J.P., Nauwelaers B., Stevens N. A survey on multiple access Visible Light Positioning. Proceedings of the 2016 IEEE International Conference on Emerging Technologies and Innovative Business Practices for the Transformation of Societies (EmergiTech).

[14] Belmonte-Fernández S., Puertas-Cabedo A., Torres-Sospedra J., Montoliu-Colás R., Trilles-Oliver S. (2017). An Indoor Positioning System Based on Wearables for Ambient-Assisted Living. Sensors.

[15] Raspopoulos M., Laoudias C., Kanaris L., Kokkinis A., Panayiotou C., Stavrou S. 3D Ray Tracing for device-independent fingerprint-based positioning in WLANs. Proceedings of the 2012 9th Workshop on Positioning, Navigation and Communication.

[16] Kanaris L., Kokkinis A., Fortino G., Liotta A., Stavrou S. (2016). Sample Size Determination Algorithm for fingerprint-based indoor localization systems. Comput. Netw..

[17] Kjærgaard M. A Taxonomy for Radio Location Fingerprinting. Proceedings of the Third International Symposium: Location and Context-Awareness.

[18] Lausnay S.D., Strycker L.D., Goemaere J.P., Stevens N., Nauwelaers B. Optical CDMA codes for an indoor localization system using VLC. Proceedings of the 2014 3rd International Workshop in Optical Wireless Communications (IWOW).

[19] Honkavirta V., Perala T., Ali-Loytty S., Piche R. A comparative survey of WLAN location fingerprinting methods. Proceedings of the 6th Workshop on Positioning, Navigation and Communication (WPNC).

[20] Kanaris L., Kokkinis A., Raspopoulos M., Liotta A., Stavrou S. Improving RSS fingerprint-based localization using directional antennas. Proceedings of the The 8th European Conference on Antennas and Propagation (EuCAP).

[21] Kokkinis A., Raspopoulos M., Kanaris L., Liotta A., Stavrou S. Map-aided fingerprint-based indoor positioning. Proceedings of the 2013 IEEE 24th Annual International Symposium on Personal, Indoor, and Mobile Radio Communications (PIMRC).

[22] Li B., Salter J., Dempster A.G., Rizos C. Indoor positioning techniques based on wireless LAN. Proceedings of the First IEEE International Conference on Wireless Broadband and Ultra Wideband Communications.

[23] Bahl P., Padmanabhan V. RADAR: An in-building RF-based user location and tracking system. Proceedings of IEEE INFOCOM 2000 Conference on Computer Communications and the Nineteenth Annual Joint Conference of the IEEE Computer and Communications Societies.

[24] Yeung W., Zhou J., Ng J. Enhanced Fingerprint-Based Location Estimation System in Wireless LAN Environment. Proceedings of the International Conference on Embedded and Ubiquitous Computing.

[25] Saha S., Chaudhuri K., Sanghi D., Bhagwat P. Location determination of a mobile device using IEEE 802.11b access point signals. Proceedings of the IEEE Wireless Communications and Networking.

[26] Nuño-Barrau G., Páez-Borrallo J.M. (2006). A New Location Estimation System for Wireless Networks Based on Linear Discriminant Functions and Hidden Markov Models. EURASIP J. Adv. Signal Process..

[27] Kemppi P., Nousiainen S. Database Correlation Method for Multi-System Positioning. Proceedings of the 2006 IEEE 63rd Vehicular Technology Conference.

[28] Youssef M., Agrawala A. The Horus WLAN location determination system. Proceedings of the 3rd International Conference on Mobile Systems, Applications, and Services.

[29] Roos T., MyllymAki P., Tirri H., Misikangas P., SievAnen J. (2002). A Probabilistic Approach to WLAN User Location Estimation. Int. J. Wirel. Inf. Networks.

[30] Evennou F., Marx F., Novakov E. Map-aided indoor mobile positioning system using particle filter. Proceedings of IEEE Wireless Communications and Networking Conference.

[31] Deng Z.A., Wang G., Qin D., Na Z., Cui Y., Chen J. (2016). Continuous Indoor Positioning Fusing WiFi, Smartphone Sensors and Landmarks. Sensors.

[32] Bluetooth Technology Website. https://www.bluetooth.com/.

[33] iBeacon—Apple Developer. https://developer.apple.com/ibeacon/.

[34] Fard H.K., Chen Y., Son K.K. Indoor positioning of mobile devices with agile iBeacon deployment. Proceedings of 2015 IEEE 28th Canadian Conference on Electrical and Computer Engineering (CCECE).

[35] Gowrishankar S., Madhu N., Basavaraju T.G. Role of BLE in proximity based automation of IoT: A practical approach. Proceedings of the 2015 IEEE Recent Advances in Intelligent Computational Systems (RAICS).

[36] Chandel V., Ahmed N., Arora S., Ghose A. InLoc: An end-to-end robust indoor localization and routing solution using mobile phones and BLE beacons. Proceedings of the 2016 International Conference on Indoor Positioning and Indoor Navigation (IPIN).

[37] Antevski K., Redondi A.E.C., Pitic R. A hybrid BLE and Wi-Fi localization system for the creation of study groups in smart libraries. Proceedings of the 2016 9th IFIP Wireless and Mobile Networking Conference (WMNC).

[38] Peng Y., Fan W., Dong X., Zhang X. An Iterative Weighted KNN (IW-KNN) Based Indoor Localization Method in Bluetooth Low Energy (BLE) Environment. Proceedings of the 2016 Intl IEEE Conferences on Ubiquitous Intelligence Computing, Advanced and Trusted Computing, Scalable Computing and Communications, Cloud and Big Data Computing, Internet of People, and Smart World Congress (UIC/ATC/ScalCom/CBDCom/IoP/SmartWorld).

[39] Kriz P., Maly F., Kozel T. (2016). Improving Indoor Localization Using Bluetooth Low Energy Beacons. Mob. Inf. Syst..

[40] TruNET Wireless. www.fractalnetworx.com.

[41] Stavrou S., Saunders S. Review of constitutive parameters of building materials. Proceedings of the Twelfth International Conference on Antennas and Propagation.

[42] Jemai J., Piesiewicz R., Kurner T. Calibration of an indoor radio propagation prediction model at 2.4 GHz by measurements of the IEEE 802.11b preamble. Proceedings of the 2005 IEEE 61st Vehicular Technology Conference.

[43] Gwon Y., Jain R. Error Characteristics and Calibration-free Techniques for Wireless LAN-based Location Estimation. Proceedings of the Second International Workshop on Mobility Management & Wireless Access Protocols.

[44] Chintalapudi K., Padmanabha Iyer A., Padmanabhan V.N. Indoor Localization Without the Pain. Proceedings of the Sixteenth Annual International Conference on Mobile Computing and Networking.

[45] Lim H., Kung L.C., Hou J.C., Luo H. (2010). Zero-configuration Indoor Localization over IEEE 802.11 Wireless Infrastructure. Wirel. Netw..

[46] Rai A., Chintalapudi K.K., Padmanabhan V.N., Sen R. Zee: Zero-effort Crowdsourcing for Indoor Localization. Proceedings of the 18th Annual International Conference on Mobile Computing and Networking.

[47] Yang Z., Wu C., Liu Y. Locating in Fingerprint Space: Wireless Indoor Localization with Little Human Intervention. Proceedings of the 18th Annual International Conference on Mobile Computing and Networking.

[48] Yang Z., Wu C., Liu Y., Xi W. (2013). WILL: Wireless Indoor Localization without Site Survey. IEEE Trans. Parallel Distrib. Syst..

